# Image-Based 3D Characterization of Abdominal Aortic Aneurysm Deformation After Endovascular Aneurysm Repair

**DOI:** 10.3389/fbioe.2019.00267

**Published:** 2019-11-01

**Authors:** Karen López-Linares, Inmaculada García, Ainhoa García, Camilo Cortes, Gemma Piella, Iván Macía, Jérôme Noailly, Miguel A. González Ballester

**Affiliations:** ^1^Vicomtech Foundation, San Sebastián, Spain; ^2^Bioengineering Area, Biodonostia Health Research Institute, San Sebastián, Spain; ^3^BCN Medtech, Department of Information and Communication Technologies, Universitat Pompeu Fabra, Barcelona, Spain; ^4^Donostia University Hospital, San Sebastián, Spain; ^5^ICREA, Barcelona, Spain

**Keywords:** abdominal aortic aneurysm, strain analysis, biomechanics, deformation, prognosis, follow-up, computed tomography angiography, biomarker

## Abstract

An abdominal aortic aneurysm (AAA) is a focal dilation of the abdominal aorta, that if not treated, tends to grow and may rupture. The most common treatment for AAAs is the endovascular aneurysm repair (EVAR), which requires that patients undergo Computed Tomography Angiography (CTA)-based post-operative lifelong surveillance due to the possible appearance of complications. These complications may again lead to AAA dilation and rupture. However, there is a lack of advanced quantitative image-analysis tools to support the clinicians in the follow-up. Currently, the approach is to evaluate AAA diameter changes along time to infer the progress of the patient and the post-operative risk of AAA rupture. An increased AAA diameter is usually associated with a higher rupture risk, but there are some small AAAs that rupture, whereas other larger aneurysms remain stable. This means that the diameter-based rupture risk assessment is not suitable for all the cases, and there is increasing evidence that the biomechanical behavior of the AAA may provide additional valuable information regarding the progression of the disease and the risk of rupture. Hence, we propose a promising methodology for post-operative CTA time-series registration and subsequent aneurysm biomechanical strain analysis. From these strains, quantitative image-based descriptors are extracted using a principal component analysis of the tensile and compressive strain fields. Evaluated on 22 patients, our approach yields a mean area under the curve of 88.6% when correlating the strain-based quantitative descriptors with the long-term patient prognosis. This suggests that the strain information directly extracted from the CTA images is able to capture the biomechanical behavior of the aneurysm without relying on finite element modeling and simulation. Furthermore, the extracted descriptors set the basis for possible future imaging biomarkers that may be used in clinical practice. Apart from the diameter, these biomarkers may be used to assess patient prognosis and to enable informed decision making after an EVAR intervention, especially in difficult uncertain cases.

## 1. Introduction

An abdominal aortic aneurysm (AAA) is a pathological weakening and ballooning of the abdominal aorta. The abdominal aorta is considered aneurismatic when its diameter is larger than 3.0 cm or when it exceeds its normal diameter by more than 50% (Sidawy and Perler, 2018). Without treatment, the aneurysm tends to grow since it is unable to withstand the forces of the blood pressure, resulting in progressive dilation, and eventually, rupture with a high mortality rate (Hoornweg et al., 2008).

Lately, the treatment of abdominal aortic aneurysms has shifted from open surgery to a minimally invasive alternative known as Endovascular Aneurysm Repair (EVAR). EVAR involves the deployment and fixation of an endograft using a catheter, introduced through the femoral arteries. This procedure excludes the damaged aneurysm wall from blood circulation, creating an intraluminal thrombus that shrinks if the intervention is successful. Even if EVAR has shown great benefits with respect to open surgery, the long-term survival rates in both approaches are almost equivalent (Stather et al., 2013). While open repair removes the aneurysm and replaces the aortic wall with a stent-graft, in EVAR the aneurysm is excluded from blood circulation but it is not removed. In the long term, this can cause EVAR procedure-specific complications that may result in post-operative aneurysm expansion and increased risk of AAA rupture, eventually leading to reintervention (Chaikof et al., 2018). Thus, patients treated with EVAR undergo lifelong surveillance to ensure the continued success of the intervention and to prevent late post-operative AAA rupture and aneurysm-related death.

The gold standard imaging technique for follow-up after EVAR is Computerized Tomography Angiography (CTA), typically performed 1 and 6 months after the intervention, and then at least yearly (Chaikof et al., 2018). Currently, the clinical procedure to assess the progress and prognosis of a patient after EVAR consists in examining CTA scans to detect potential complications or an aneurysm enlargement (unfavorable evolution). The most common type of post-EVAR complications are grouped under the term endoleaks and refer to the persistent blood-flow within the excluded aneurysm, which may again pressurize the AAA and increase the risk of rupture (Chaikof et al., 2018). AAA enlargement can happen due to the presence of complications visible in the CTA at the time the image is acquired or with no discernible complication, generally termed as endotension, which causes clinical doubts on how to proceed with the post-operative treatment of the patients (Chaikof et al., 2018).

The size of the aneurysm, determined by its maximum diameter measured at a certain slice, is used to evaluate the progression of the AAA and its risk of rupture post-operatively. However, there is a lack of standardization in terms of determining the degree and rate of disease progression and a significant variability exists when measuring and reporting the diameter value (Chaikof et al., 2018). Furthermore, several works have reported that there is a significant number of small AAAs that progress to rupture, whereas many larger AAAs remain stable. This suggests that AAA size may not be the only predictor of AAA rupture (Nicholls et al., 1998; Lederle et al., 2002; Kolipaka et al., 2016; Salman et al., 2019).

There is an increasing evidence that other variables, such as AAA volume or morphological changes, or the variance of biomechanical factors along time, could provide valuable information to analyze and predict the behavior of the treated aneurysm and the prognosis of the patient (Sun, 2012). However, this information is currently not evaluated in the clinical routine due to the lack of advanced quantitative image analysis tools to support the clinician during the follow-up of patients.

Hence, the aim of this work is to propose and evaluate a promising methodological approach to aid the clinician in the post-operative surveillance of patients, by providing an image analysis pipeline to characterize aneurysm deformation and to correlate it with the long-term treatment prognosis. The proposed pipeline is based on the registration of 3D post-operative CTA scans to compute displacement fields, followed by the analysis of those fields to extract the actual biomechanical strains and to correlate them with the patient prognosis, which sets the basis for future imaging biomarkers that could be used in clinical practice.

Preliminary studies have shown that the 3D-3D alignment of CTA images is feasible (Demirci et al., 2009; Matl et al., 2017), yet the goal of those studies was to evaluate aortic changes due to the placement of the endograft, without analyzing or quantifying the long-term deformations of the aneurysm. In Maiora et al. (2010), a 3D-3D post-operative CTA registration approach for thrombus change visualization was introduced, but no quantitative analysis of those changes was provided. On the other hand, multiple studies in the literature address aneurysm biomechanics based on mathematical and computational models (Raut et al., 2013; Chauhan et al., 2017). They commonly evaluate peak wall stresses, which they associate to the aneurysm rupture risk in the pre-operative scenario (Indrakusuma et al., 2016; Leemans et al., 2017). Other works quantify the biomechanics from *in-vivo* magnetic resonance elastography images to study the correlation between the diameter and the AAA stiffness (Kolipaka et al., 2016). Our approach is different from previous works, since we evaluate the aneurysm strain directly from information extracted from the CTA images without relying on complex finite element modeling and simulations. To the best of our knowledge, this is the first approach to aneurysm strain analysis in the post-operative scenario, correlating the deformation fields with the prognosis of the patient. This information may be valuable for the clinicians to better evaluate the post-operative progression of the AAA and its risk of rupture, especially in difficult uncertain cases. It may also reflect the presence of endoleaks not discernible at the time the CTA scan is taken, such as in endotension cases.

The outline of the paper is as follows: in section 2 the employed CTA imaging datasets are described, as well as the segmentation methods used to extract each structure that is needed as input for the proposed approach. The steps for the registration of post-operative time series and the subsequent computation and quantification of the mechanical strains are also presented in section 2. In section 3 the experiments and results are summarized, and finally, section 4 presents some discussion and concluding remarks.

## 2. Materials and Methods

This section describes the employed datasets and the proposed approach to extract the post-operative mechanical strains, to correlate them with the patient prognosis. In summary, our method has the following steps:

1. Segmentation of CTA datasets using previously developed algorithms.
2. Rigid registration of the vertebrae and hips to spatially align post-operative datasets from the same patient.
3. Straightened Curved Planar Reformatting (CPR) along the aortic centerline to remove displacements of the aorta.
4. Straightened CPR-based deformable registration to evaluate changes intrinsic to the thrombus or aneurysm.
5. Displacement field-based strain analysis.

### 2.1. CTA Datasets

Our experiments are run on 44 post-operative CTA scans of 22 patients treated with the same standard EVAR technique at Donostia University Hospital (Spain). The age of the patients ranged from 63 to 87 years, and 21 of them were male. Ethical approval was obtained for the current study by the “*Comité Ético de Investigación Clínica del Área Sanitaria de Gipuzkoa*.”

**Figure 1:**
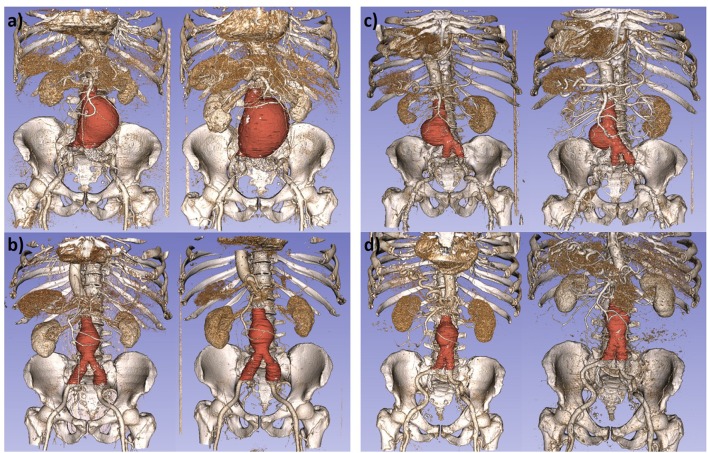
Volume renderings of computed tomography angiography scans, with superimposed aneurysm segmentations for some patients in our study. **(a,b)** Images of patients with unfavorable evolution at t1 (left) and t2 (right). **(c,d)** Images of patients with favorable evolution at t1 (left) and t2 (right).

Protocols for EVAR surveillance usually establish the need of a CTA scan at 1, 6, and 12 months after initial repair, but the 6-month scan can be eliminated from surveillance if the 1-month scan shows no concerning endoleak or sac enlargement (Chaikof et al., 2018). Thus, in the current work, the 1-month and the 12-month scans are employed, since these scans are available for all the patients. Nine patients in our database had a long-term unfavorable evolution: 5 of them were re-intervened, 1 of them had a ruptured AAA, and 3 of them are under post-operative surveillance due to high surgical risk. Figure 1 displays volume renderings of CTA scans of unfavorable (Figures 1a,b) and favorable (Figures 1c,d) evolution patients at the two time points. For the unfavorable evolution cases an expansion of the AAA can be observed.

The employed datasets were obtained with scanners of different manufacturers with an in-plane spatial resolution in the range of 0.725–0.977 mm in x and y, and a slice thickness from 0.625 to 1 mm in the z direction.

### 2.2. Segmentation of CTA Datasets

In order to align and compare the two post-operative CTA scans of each patient, our proposed pipeline requires the segmentation of the vertebrae and hip, the aortic lumen, and the aneurysm, as well as the extraction of the aortic centerline.

#### 2.2.1. Vertebrae and Hip Segmentation

Vertebrae and hip segmentation is essential since the proposed pipeline starts from the rigid alignment of these structures. In a CTA image, the bones appear with a high intensity, which allows coarsely segmenting them with simple thresholding operations. As the goal is to have an initial alignment, a segmentation using a connected components analysis of the thresholded image is enough as input for the pipeline.

#### 2.2.2. Lumen Segmentation and Centerline Extraction

The aortic lumen appears in the image as a highly contrasted structure with marked borders, due to the injected contrast agent and the presence of the metallic stent. To segment it, the adaptive region growing algorithm proposed in Macía et al. (2016) is employed.

**Figure 2:**
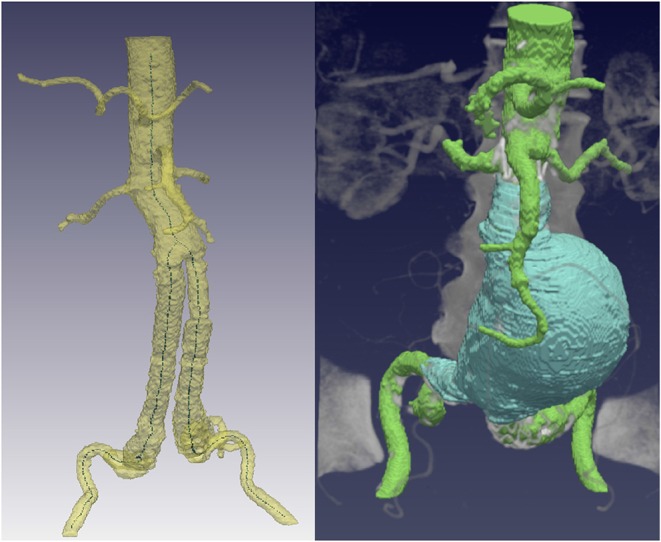
Required segmentations that serve as input for the proposed alignment pipeline. **(Left)** Aortic lumen segmentation and centerline extraction, keeping only the three main branches. **(Right)** Consistently segmented aneurysm, starting from the lowest renal to the iliac bifurcation.

In order to obtain the centerline, an initial skeleton is extracted from the segmented lumen via distance-based homotopic thinning. This skeleton is noisy and presents spurious branches and loops that must be removed in order to have a final centerline, which is obtained following the procedure in Macía et al. (2016). Since the goal is to use the centerline to compute the straightening of the aorta, only the three largest branches are kept, which correspond to the main aortic centerline and the centerlines in the endograft-limited area, as shown in Figure 2.

#### 2.2.3. Aneurysm Segmentation

A precise aneurysm segmentation is the key for the subsequent strain analysis. It is important that it is consistently segmented within the same anatomical region in the two CTA datasets of the same patient, to avoid output deformation fields that correspond to errors when selecting the aneurysm area. Thus, the thrombus is always segmented in a region spanning from the lowest renal artery to the iliac bifurcation (see Figure 2), which are common clinical landmarks, using the convolutional neural network developed in López-Linares et al. (2018).

### 2.3. Post-operative CTA Registration Pipeline

Hereby, we propose a CTA time-series registration pipeline that enables the analysis of the deformations of the aneurysm post-operatively to correlate them with the long-term outcome of a patient treated with EVAR. However, the comparison of time-series is hindered by patient position changes and aortic deformations and displacements, which should be removed before evaluating the intrinsic aneurysm changes. Thus, the developed registration pipeline has three main steps: (1) a landmark-based rigid registration of the vertebrae and hips to eliminate patient position and orientation changes, (2) straightened curved planar reformation of the aorta to remove aortic displacements, since blood vessels are not rigid structures, and (3) deformable registration of the thrombus to capture changes intrinsic to the aneurysm. These steps are further explained in the following subsections.

#### 2.3.1. Rigid Registration of the Vertebrae and Hip

**Figure 3:**
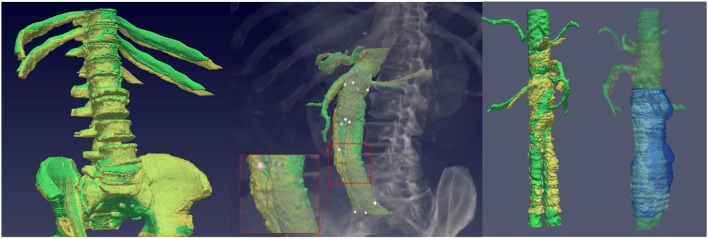
**(Left)** Rigid registration of the vertebrae and co-registration of the aortic lumen. A displacement in the aortic lumen between the reference scan (green) and the co-registered lumen (yellow) is observed in the zoomed region. **(Right)** Lumen and thrombus straightened curved planar reformations from the two scans of a patient.

The proposed registration methodology starts from the rigid alignment of the segmented vertebrae and hip, since they are the most rigid structures that appear in abdominal CTA datasets. The registration of the two post-operative datasets of each patient is initialized with a landmark-based translation that roughly brings the second CTA closer to the first post-operative scan. The lowest renal artery position, previously employed for the aneurysm segmentation, is employed as landmark, allowing optimization of registration time and ensuring convergence. Then, a rigid 3D registration procedure is performed (Johnson et al., 2015), which finds the appropriate rotations and translations to fully align the vertebrae and hip from both scans by minimizing a simple intensity-based registration metric. Figure 3 shows the output of the vertebrae rigid registration process for a sample dataset, where a displacement in the aortic lumen is observed after co-registration, since the aorta is not a rigid structure.

#### 2.3.2. Straightened Curved Planar Reformation

Since the final goal is to study the intrinsic deformations of the aneurysm, it is necessary to remove the displacements suffered by the aorta as they introduce errors when evaluating the final deformation fields. Hence, the second step of the pipeline consists in applying a centerline-based straightened Curved Planar Reformation (CPR) to the lumen and thrombus of each dataset.

In vascular image analysis, one way to display vessels is to generate longitudinal cross-sections at each centerline point in order to show their lumen, aneurysm, and surrounding tissues (Kanitsar et al., 2002). This allows the whole length of the tubular structure to be displayed within a single image, which is known as CPR. The straightened CPR generates a linear representation of the vessel centered on the actual centerline and with varying diameter. This linear representation of the aortic lumen eliminates the aortic displacements, allowing to study the actual deformations of the aneurysm.

Given the registered centerline, the CPR resamples it and casts lines perpendicular to it until the whole extent of the centerline is swept. In the extracted post-operative centerlines three branches are observed, which correspond to the lumen areas constrained by the main endograft module and the two submodules for the bifurcation, as can be seen in Figure 1. Since a unique centerline branch is required for the CPR, we emulate a combined lumen in the region restrained by the two endograft submodules and extract the center point from both branches (see Figure 2). Then, we resample the centerline equidistantly with a sampling distance of 1 mm and a sagittal projection direction, which results in slices that have a height equal to the length of this sampled centerline. This procedure is repeated for the two post-operative CTA scans of each patient, as shown in Figure 3.

Owing to possible differences in the centerlines extracted from each dataset of a patient, the output straightened CPR volumes showing the aortic lumen and aneurysm may have a different size. Thus, the final step consists in applying a translation-based registration of the straightened CPR volumes. We apply the same straightened CPR to the lowest renal anatomical landmark from the previous steps and utilize it to align both volumes.

#### 2.3.3. Deformable Registration of the Thrombus

The final step of the registration pipeline consists in the computation of the displacements fields, that will be subsequently utilized to analyze the aneurysm strain and to correlate it with the evolution of the patient. These fields, which represent how the aneurysm at the second time point has changed with respect to the first post-operative scan, are obtained from the deformable registration of the straightened thrombus volumes.

**Figure 4:**
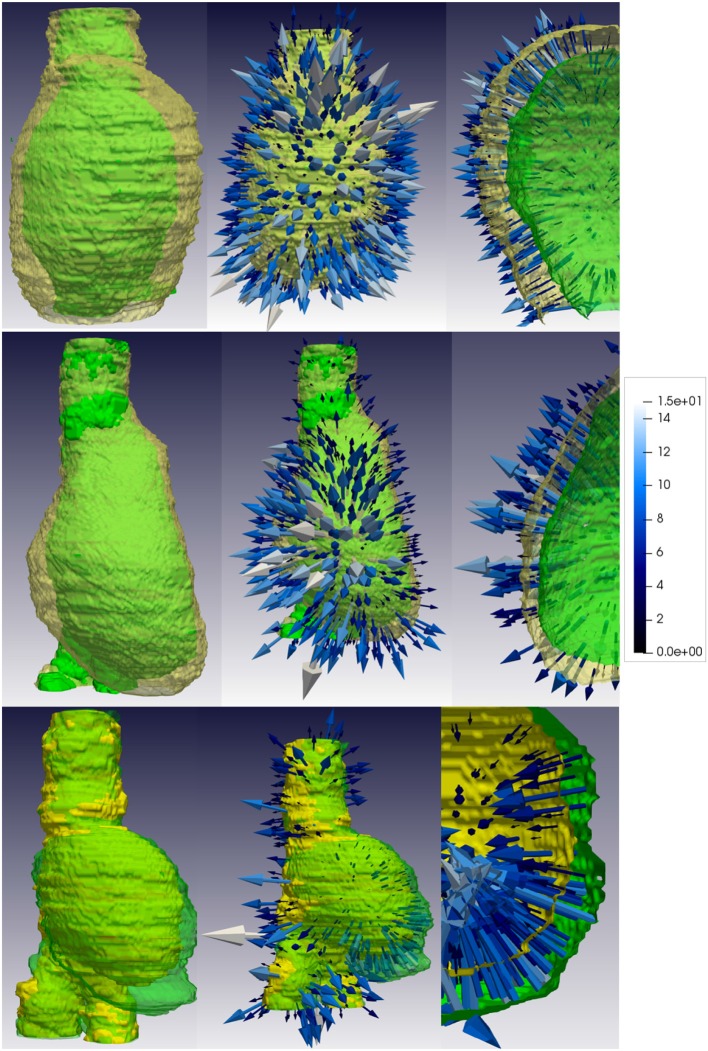
Displacement fields produced by the proposed pipeline for two unfavorable evolution patients (top and middle rows) and a favorable evolution patient (bottom row). The first column depicts the straightened thrombi that serve as input to the deformable registration step, where the reference case is shown in green and the thrombus at the second time point is shown in yellow. The second column represents the obtained displacement fields, whereas the third column corresponds to a zoomed region. For unfavorable evolution cases (first and second rows) most arrows point toward the outside of the aneurysm, while for favorable evolution cases arrows generally point toward the inside, representing an shrinkage of the aneurysm. The color of the arrows represents the magnitude of the displacement. The vectors are discretized and scaled by their displacement magnitude for visualization purposes.

The level-set motion algorithm proposed in Vemuri et al. (2003) is applied, which relies on a curve evolution approach expressed in a level-set framework to achieve image intensity morphing simply and efficiently. The basic idea is to let the aneurysm s-CPR of the second CTA scan evolve during 200 iterations, by letting its level-sets move along their respective normals in order to minimize the mean square difference between both images. The speed of the evolution is proportional to the difference between the aneurysm s-CPR of the first CTA (target) and the evolving aneurysm of the second CTA scan (source), until the source becomes the target image. We evaluate the final accuracy of the registration by computing the Dice similarity metric (Wu et al., 2019) between the reference image and the registered image, which yields a high mean Dice similarity coefficient of 0.975±0.012. Figure 4 depicts some examples of the output fields produced by the proposed registration pipeline.

### 2.4. Displacement Field-Based Strain Analysis

The computed displacement fields relate the configuration of each aneurysm at the second time-point with its corresponding undeformed reference configuration at time-point one. Thus, in the Lagrangian description, the displacement field U of the points of the aneurysm between the reference and the second time-point configurations can be expressed as:

(1) $$\begin{array}{l} \underline{U}(\underline{P_{0}},t)=\underline{P_{1}}(\underline{P_{0}},t)-\underline{P_{0}}\\ \;=\underline{U}(x,y,z,t)=\underline{P_{1}}(\underline{P_{0}}(x,y,z),t)-\underline{P_{0}}(x,y,z) \end{array}$$

where *P*_1_ are the points that characterize the aneurysm at the second post-operative scan *t* seen from the reference configuration, and *P*_0_ are the points that characterize the aneurysm at the reference post-operative scan. The deformation tensor $\underline{\underline{F}}$*(x,y,z,t)* at each aneurysm voxel can then be computed from the displacement gradient field as follows:

(2) $$\begin{array}{l} \underline{\underline{F}}(x,y,z,t)=\underline{\underline{F}}\left(\underline{P_{0}},t\right)=\frac{\partial \underline{U}(\underline{P_{0}}(x,y,z),t)}{\partial \underline{P_{0}}(x,y,z)}+\underline{\underline{I}}\\ \;=\left(\begin{array}{c} \frac{\partial U_{x}}{\partial P_{0x}}\frac{\partial U_{x}}{\partial P_{0y}}\frac{\partial U_{x}}{\partial P_{0_{z}}}\\ \frac{\partial U_{y}}{\partial P_{0x}}\frac{\partial U_{y}}{\partial P_{0y}}\frac{\partial U_{y}}{\partial P_{0_{z}}}\\ \frac{\partial U_{z}}{\partial P_{0_{x}}}\frac{\partial U_{z}}{\partial P_{0y}}\frac{\partial U_{z}}{\partial P_{0_{z}}} \end{array}\right)+\underline{\underline{I}} \end{array}$$

where $\underline{\underline{I}}$ is the identity tensor. Central numerical differentiation is employed to compute the derivatives from U.

Assuming a finite strain regime, the Lagrangian deformations of the aneurysms can be described by means of the right Cauchy-Green strain tensor, calculated at each voxel (i.e., point) as:

(3) $$\underline{\underline{C}}={\underline{\underline{F}}}^{T}\underline{\underline{F}}.$$

The principal components of the right Cauchy-Green strain tensor are the squares of the principal stretches. More specifically, the largest eigenvalue of $\underline{\underline{C}}$ corresponds to the maximum tensile strain when the corresponding principal stretch is larger than 1. In turn, the smallest eigenvalue corresponds to the maximum compressive strain when the corresponding principal stretch is smaller than 1. A maximum principal stretch inferior to 1 or a minimum principal stretch superior to 1 would, respectively, mean that a material point is triaxially tensed or compressed, respectively. Hence, applied to each voxel of the wall of the aneurysm our analysis indicates locally in which directions the wall volume experiences the largest deformations and the scalar results indicates whether these deformations are tensile or compressive strains. Following this procedure, we generate tensile and compressive strain unit vector images for each patient case, which characterize the distribution of the extremal strains of the aneurysm at the second time-point with respect to the first time-point.

The final step of our method consists in quantitatively analyzing the extracted tensile and compressive strain fields to associate these descriptors with patient prognosis. Hence, it is necessary to define image metrics that summarize the global appearance of the tensile and compressive strain vectors. We hypothesize that the principal component analysis (PCA) of the fields can capture whether each field is globally tangential or normal to the wall, so as to achieve quantitative image-based descriptors that are able to differentiate between favorable and unfavorable evolution patients. PCA is an essential tool in shape modeling, which allows to sharply reduce the dimensionality of the strain data, maintaining the most relevant information, and eliminating noise. This property is essential to discard the information from deformation vectors produced due to small errors in the segmentation or registration process. PCA identifies directions (i.e., principal components) along which the variation in the data is maximal. This property allows us gain physical insight into dominant deformation patterns.

Hence, we compute the PCA for each tensile and compressive strain field, and we obtain 3 principal components per field. These components define the principal directions of each field and can be used to perform a Receiver Operating Characteristic-Area Under the Curve (ROC-AUC) analysis. To test that in fact these principal components capture the global pattern of the strain fields and for analytic purposes, we train a linear Support Vector Machine (SVM) classifier to separate between favorable and unfavorable evolution cases. An SVM is a supervised machine learning method capable of deciphering subtle patterns in noisy and complex datasets (Gholami and Fakhari, 2017). The idea is to identify a hyperplane that separates the datasets in two classes (favorable and unfavorable evolution) from some input features. We run three experiments using as features: (1) principal components of tensile fields (9 tensile features per patient, corresponding to the first 3 principal components); (2) principal components of compressive fields (9 compressive features per patient, corresponding to the first 3 principal components); and (3) principal components of tensile and compressive fields together (18 features per patient). A four-fold stratified cross-validation approach is followed, splitting the data into validation and train sets by preserving the percentage of samples for each evolution class. To provide more robustness to our analysis, this four-fold cross-validation is run 10 times, and the mean ROC-AUC curve is computed.

## 3. Results

The proposed methodology is applied to the 22 patients in our database, for whom, first, the displacement fields are extracted and analyzed. Figure 4 represents these displacement fields at discrete points for 2 patients with unfavorable evolution, and a patient with favorable evolution. The reference surface, i.e., the straightened CPR of the aneurysm of the first time-point scan, is displayed in green color. The displacement fields describe how the aneurysm from the first time point has evolved into the thrombus from the second scan. The arrow tip points toward the direction of the displacement: an arrow pointing from the surface toward the inside of the aneurysm represents a shrinkage in that region, whereas an arrow pointing outside describes an aneurysm expansion. The color of the arrow and its length depict the magnitude of the displacement. Based on a qualitative evaluation of Figure 4, it is noticeable that for patients with an unfavorable evolution most of the arrows point outwards, while for those cases with a good evolution the strongest displacements point inwards. These arrows have been magnified for visualization purposes.

**Figure 5:**
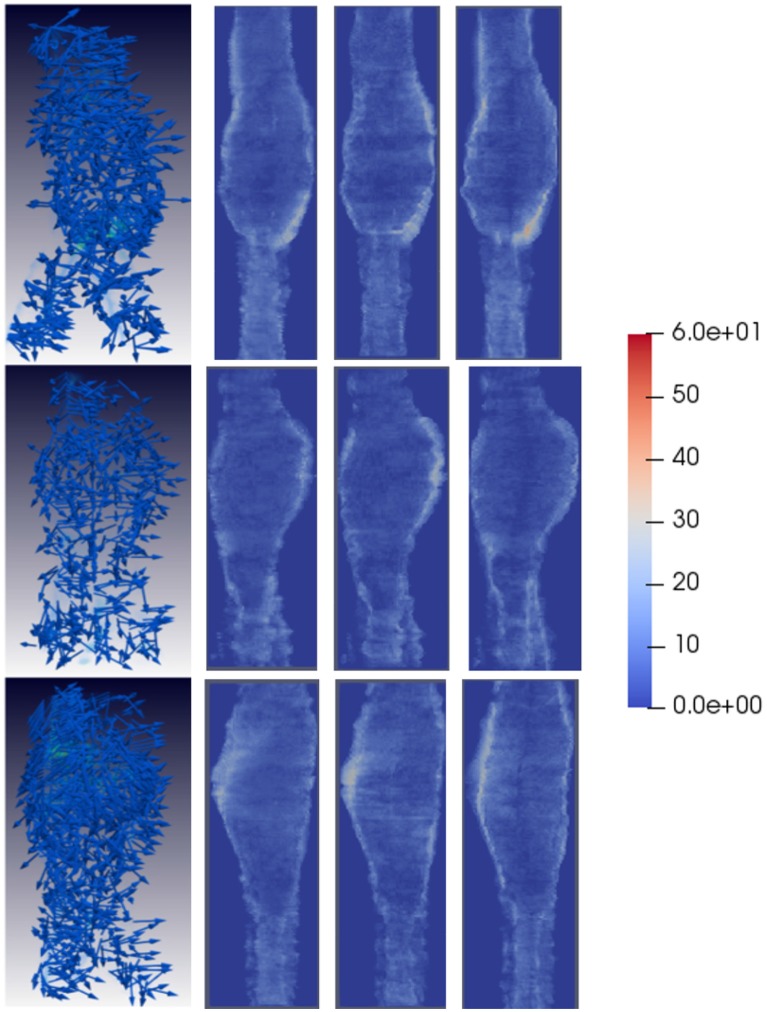
Tensile strain patterns for some example favorable evolution patients. The first column shows tensile strain fields for three different patients, where the tensile strain vectors are randomly oriented. The second, third, and fourth columns depict the magnitude of the vector components in each direction, projected into the main AAA axis to show the dominant direction of the strain patterns. For favorable evolution cases no dominant direction is observed, represented by the lack of red areas, which coincides with the conclusion that the strain vectors in these cases are randomly oriented. Vectors have been sampled every 100 points for visualization purposes.

**Figure 6:**
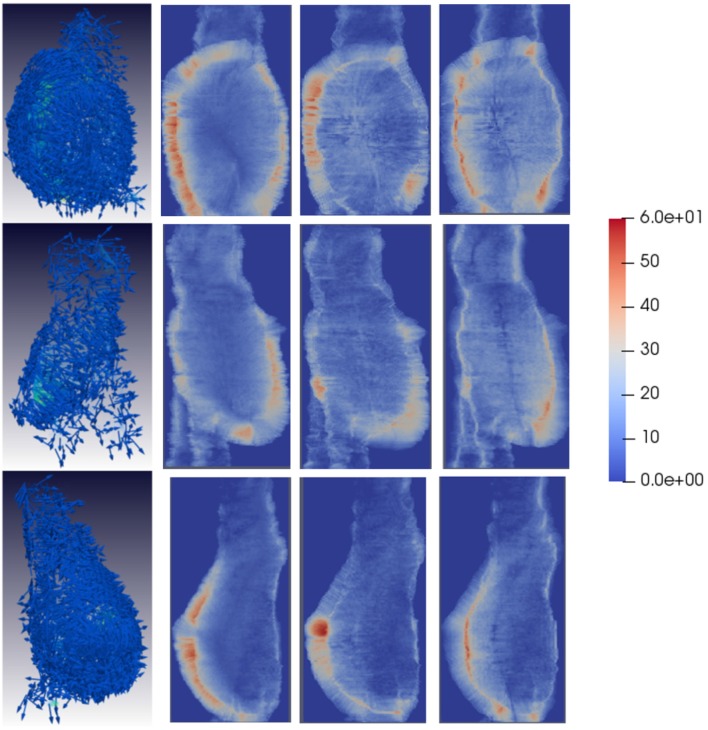
Tensile strain patterns for some example unfavorable evolution patients. The first column shows tensile strain fields for three different patients. In unfavorable evolution cases, the tensile vectors are mostly tangential to the AAA surface. The second, third, and fourth columns depict the magnitude of the vector components in each direction, projected into the main AAA axis to show the dominant direction of the strain patterns. For unfavorable evolution cases the dominant directions can be clearly observed, which are represented by reddish areas mostly in the region the AAA is expanding. Vectors have been sampled every 100 points for visualization purposes.

**Figure 7:**
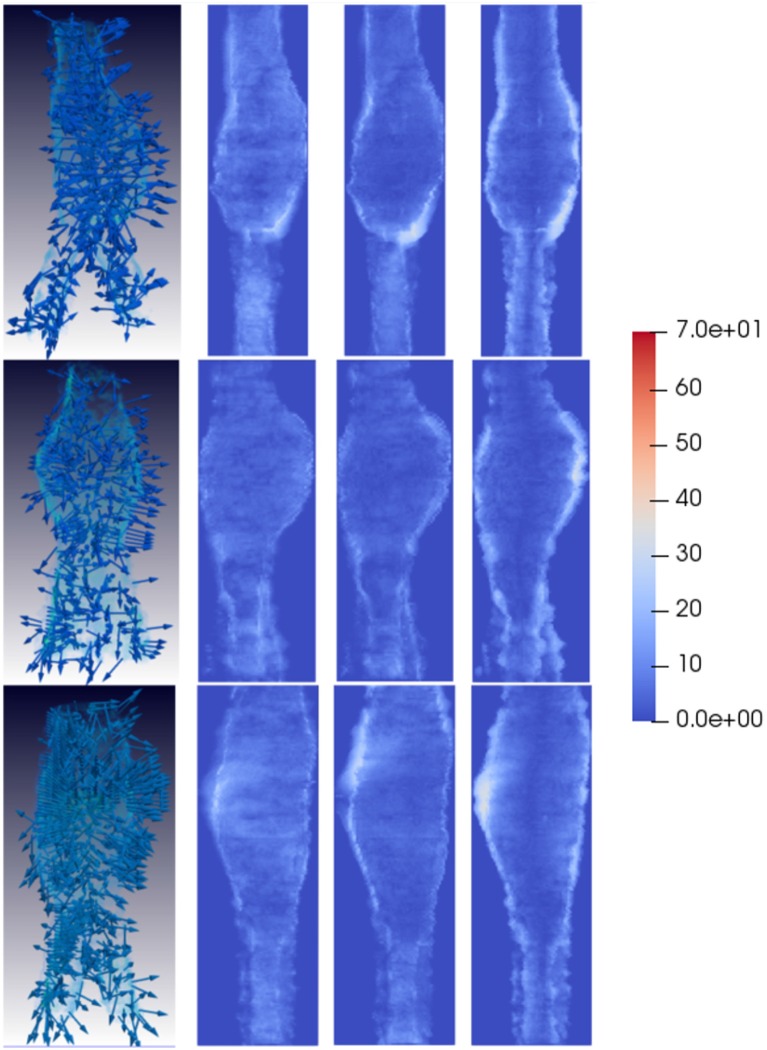
Compressive strain patterns for some example favorable evolution patients. The first column shows compressive fields for three different patients, where the vectors are randomly oriented. The second, third, and fourth columns depict the magnitude of the vector components in each direction, projected into the main AAA axis to show the dominant direction of the strain patterns. For favorable evolution cases no dominant direction is observed, represented by the lack of red areas, which coincides with the conclusion that the strain vectors in these cases are randomly oriented. Vectors have been sampled every 100 points for visualization purposes.

**Figure 8:**
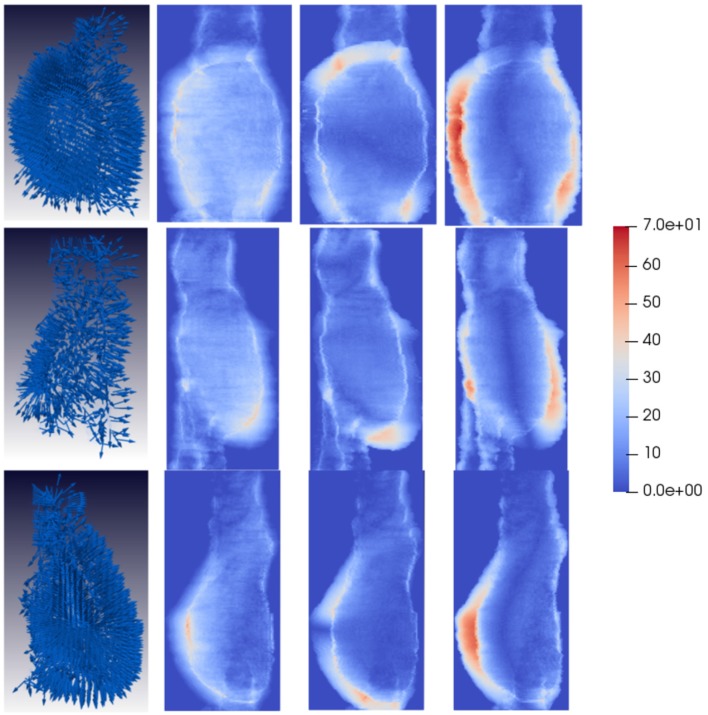
Compressive strain patterns for some example unfavorable evolution patients. The first column shows compressive fields for three different patients, where the vectors are generally oriented normally to the AAA wall forming a hedgehog-like shape. The second, third, and fourth columns depict the magnitude of the vector components in each direction, projected into the main AAA axis to show the dominant direction of the strain patterns. For unfavorable evolution cases the dominant directions can be clearly observed, which are represented by reddish areas mostly in the region the AAA is expanding. Vectors have been sampled every 100 points for visualization purposes.

**Figure 9:**
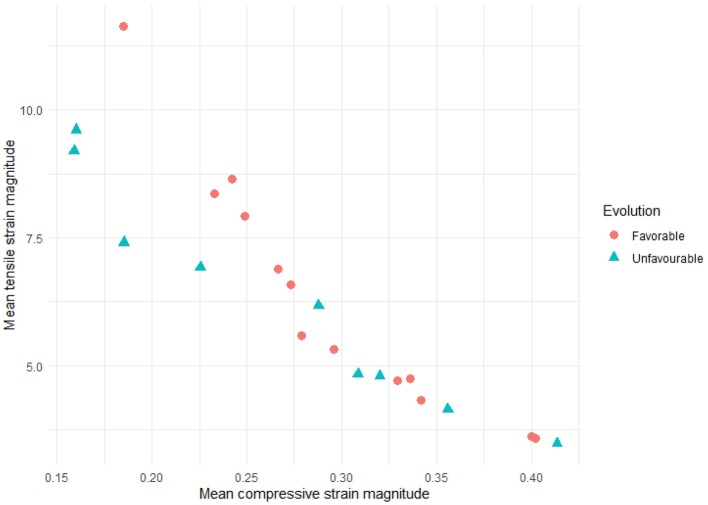
Mean tensile strain magnitudes vs. mean compressive strain magnitudes for all the patients in our study. A negative correlation between tensile and compressive strains can be observed, but no relation with patient evolution can be inferred only from the mean magnitude values.

From the displacement fields of each patient, first aneurysm deformations are computed following the strain analysis step described in section 2.4. Figures 5–8 show examples of the extracted maximum tensile and compressive fields, respectively, for favorable and unfavorable evolution patients. The displayed fields show unit vectors, since only the orientation of the vectors is considered for the analysis. Regarding the magnitude of the vectors, the mean tensile strain magnitude and the mean compressive strain magnitude per each case have been computed, which clearly show a negative correlation between them. However, there is no clear correlation between the mean strain magnitude values and the prognosis of the patient, as shown in Figure 9.

Qualitatively, looking at Figure 6 tensile vectors corresponding to unfavorable evolution cases are more tangential with respect to the surface of the aneurysm and they seem to be wrapping around the surface. On the other hand, according to Figure 8, compressive vectors for unfavorable cases are hedgehog shaped, where many vectors are normal to the surface at each point. Altogether, these particular results consistently indicate a lateral expansion of the wall, reflected by maximum tensile and compressive strains locally tangential and normal to the wall, respectively. These main directions can be further observed when independently visualizing the vector components, as shown in Figures 6, 8. The presence of red color indicates that there is a dominant direction in that region, which appears mainly in the areas in which the aneurysm is expanding. The lack of reddish areas in the favorable evolution cases coincides with the observation that strain vectors are randomly distributed for these cases, with no dominant directions.

**Figure 10:**
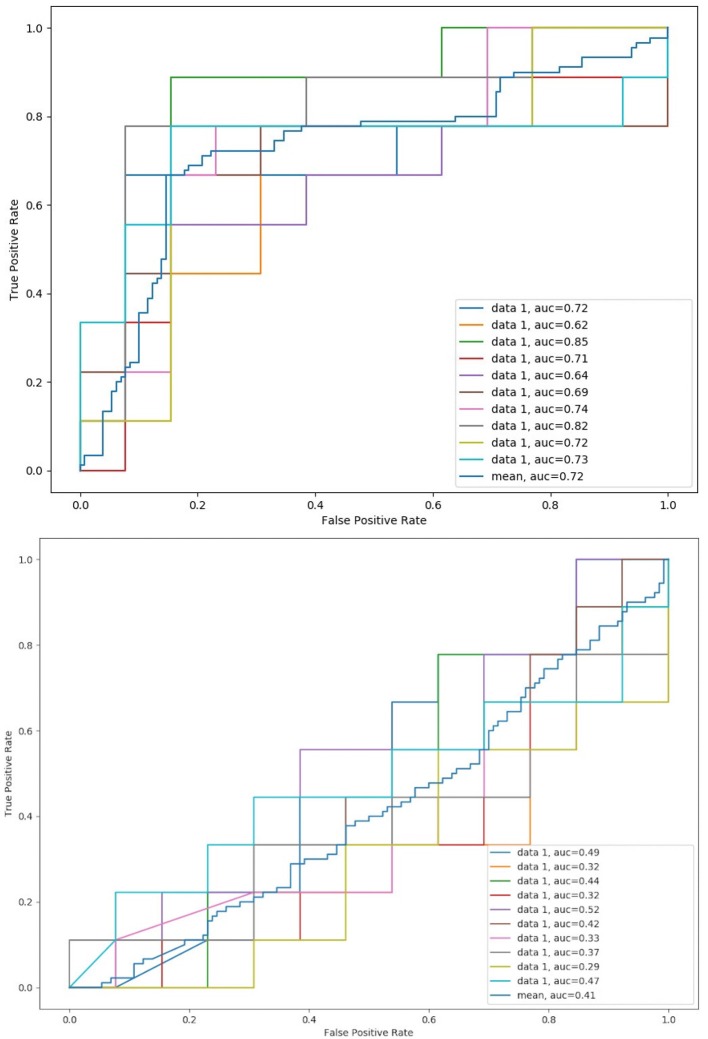
Mean ROC-AUC for all four-fold cross-validation steps and global mean for the 10 runs using only principal components from tensile strain fields **(top)** and compressive strain fields **(bottom)**.

Then, the principal components of the tensile and compressive strains are computed and used as features to train the SVM classified for analytic purposes. In the first and second experiments, the SVM classifier is trained with the tensile features alone, or with the compressive features alone, respectively. Figure 10 shows the resulting ROC-AUC curves for these two experiments. Tensile vectors alone show a good correlation with the evolution of the patient (mean AUC = 72.8%), while compressive vectors alone are not able to capture the differences between favorable evolution and unfavorable evolution cases.

**Figure 11:**
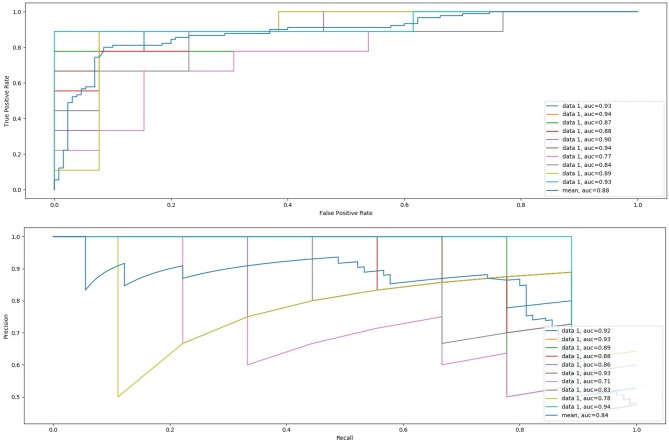
Mean ROC-AUC and precision-recall curves for all four-fold cross-validation steps and global mean for the 10 runs using tensile and compressive principal components together.

Finally, the SVM classifier is trained with all tensile and compressive features together. The ROC-AUC analysis using all the features yields a mean AUC of 88.6%, which suggest that our proposed methodology is suitable to characterize the aneurysm biomechanics directly from the image and to correlate it with patient prognosis. Figure 11 presents the obtained ROC-AUC and precision-recall curves when combining all tensile and compressive principal components at each time, as well as the global mean.

### 3.1. Comparison With Diameter-Based Progression Assessment

In order to evaluate the ability of the proposed methodology to provide additional information about the prognosis of the patient, we have automatically measured the maximum diameter of the aneurysms at both CTA scans of the same patient and calculated the difference. A diameter difference larger than 5–10 mm should prompt further evaluation and is considered clinically relevant regarding the risk of rupture, as stated in Chaikof et al. (2018) and Wanhainen et al. (2019).

**Figure 12:**
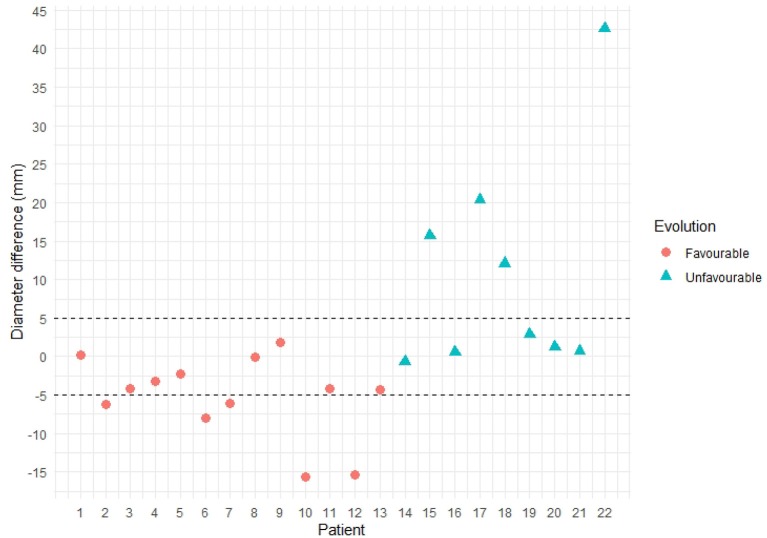
Maximum diameter difference per patient. Patients with unfavorable evolution are shown in blue, whereas patients with favorable evolution are shown in orange.

Figure 12 shows the maximum diameter differences for all the patients in this study, where it can be observed that in less than half of the cases the difference is larger than 5 mm. Furthermore, for many cases the differences are very small and close to the resolution of the images. There are also some favorable evolution cases in which the maximum diameter difference is positive, meaning that at the second CTA scan the diameter is larger than at the first time point, and unfavorable evolution cases in which the difference is negative. Hence, using only the diameter criteria these cases can be considered as uncertain, and having additional information as proposed in the current study may help better evaluating the progression of the disease.

## 4. Discussion

According to the American Society for Vascular Surgery practice guidelines on the care of patients with an abdominal aortic aneurysm (Chaikof et al., 2018), appropriate post-operative surveillance is necessary to minimize subsequent aneurysm-related death or morbidity. This Society poses the need to define imaging biomarkers of unfavorable evolution, i.e., aneurysm rupture risk, and highlights some areas of uncertainty in the care of patients with these aneurysms that would benefit from further investigation, among which finding the most effective surveillance protocol and tools to support it is emphasized.

Currently, the clinical procedure to quantitatively assess the progress and prognosis of a patient consists in measuring the 2D maximum diameter, but there is a lack of standardization in terms of determining the degree and rate of disease progression and a significant variability exists when measuring and reporting its value (Chaikof et al., 2018). Additionally, the precise mechanisms leading to AAA rupture remain unclear (Salman et al., 2019), since there are small aneurysms that rupture and larger ones that remain stable. There is an increasing evidence that other variables, such as the aneurysm morphological changes, the deformations along time and the biomechanical behavior may provide valuable information to analyze and predict the behavior of the aneurysm post-operatively (Sun, 2012).

Hereby, we have proposed a promising methodology to leverage more information contained within the post-operative CTA images of the patients to provide a better understanding of the evolution of the patient during follow up and to assist clinical decision making. The methodology starts from the registration of CTA time-series of patients with an AAA to extract the displacement fields characterizing the intrinsic changes in the aneurysm morphology during the follow-up of an EVAR intervention. From these displacement fields, a novel image-based aneurysm strain analysis is provided, which allows biomechanically characterizing the evolution of the patient by means of the computation of the aneurysm tensile and compressive strains at each voxel, extracted directly from the information in the CTA. The characterization of these tensile and compressive fields, based on a principal component analysis (PCA), allows to evaluate the biomechanical behavior of the aneurysm quantitatively, and to correlate it with the long-term prognosis of the patient.

On the one hand, our proposed registration pipeline is able to generate displacement fields that provide a meaningful representation of the aneurysm's intrinsic changes along time. To date, the comparison between time-points is mostly done by synchronizing two CTA series and concurrently visualizing them, without considering further information that could assist in detecting local changes difficult to see just by looking at the original gray-scale images. The visualization of the displacement fields, including color coded information about the magnitude and direction of the aneurysm surface deformation at each point, such as in Figure 4, provides valuable visual insight into the aneurysm evolution, allowing to assess global changes and local deformations of clinical significance, that may be indicative of pressure changes and may help clinicians gaining a better understanding of the cases.

Regarding the interpretation of these displacement fields, different patterns are observed for favorable and unfavorable evolution patients. Patients with a good evolution present fields that characterize the remodeling of the aneurysm after the intervention, in which many vectors point toward the inside of the aneurysm surface due to the shrinkage of the aneurysm. On the other hand, patients with an unfavorable evolution come out with fields where many vectors point outwards, representing the global enlargement of the aneurysm. With the available data, it is difficult to establish different deformation patterns for each endoleak type, but with a larger image database local deformations could be correlated with the location of the leaks.

On the other hand, the displacement fields and the analysis thereof through the continuum mechanics theory allow to biomechanically interpret the information extracted from CTA time-series, providing the basis for the definition of image-based biomarkers for EVAR follow-up. The eigenvectors of the right Cauchy-Green matrix represent the aneurysm tensile and compressive strain at each voxel, which again can be visualized to understand the global strains suffered by the aneurysm post-operatively. We have observed that for patients with unfavorable evolution the tensile strain fields are tangential to the surface of the aneurysm, whereas for patients with a favorable evolution these fields have a direction closer to the normal of the surface at each point. Compressive strain fields are, however, more difficult to interpret. It seems that for patients with an unfavorable evolution compressive strain fields show a more hedgehog shape, where the minimum principal strains, i.e., maximum compressive strain, within the wall have an outward direction.

**Figure 13:**
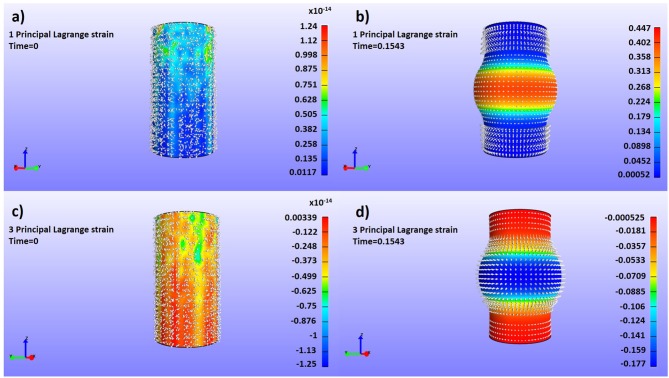
Dummy finite element simulations to assess the strain profiles of a pressured cylinder with aneurism-like deformations with superposed representations of the color map and vector fields of the maximum **(a,b)** and minimum **(c,d)** principal components of the Green-Lagrange strain tensor (Lagrangian Strain). **(a)** Maximum tractions, low internal pressure; **(b)** Maximum tractions, high internal pressure; **(c)** Maximum compressions, low internal pressure; **(d)** Maximum compressions, high internal pressure.

To gain insight into the validity of the strain patterns that we obtain for the evolution of real aneurysms, we resort to the similarity of our mechanical study with that of cylinder stress theory. To do so, we have built a dummy finite element model that simulated a soft cylinder with characteristics roughly similar to an abdominal aorta (i.e., diameter of 20 mm, 1 mm thick wall modeled as a Neo-Hookean material, and shear modulus of 0.8 MPa). For simulating an aneurysm-like inflation, an internal pressure of 0.013 MPa was applied locally to a centered segment of the tubular structure with height of 1/3 of the total cylinder length with direction normal to the cylinder face. The obtained principal Lagrangian strains (eigenvalues of the Green-Lagrange strain tensor defined as $\left(\underline{\underline{E}}=\;1/2(\underline{\underline{C}}-\underline{\underline{I}}\right)$, with $\underline{\underline{C}}$ being the right Cauchy-Green strain tensor) are shown in Figure 13. In the cylinder inflated area, the maximum principal strains tend to be oriented tangentially to the local curvature of the bulged wall (Figure 13b), and the minimum principal strains tend to be oriented in the radial direction of the bulged wall (Figure 13d). These results match with the observed orientation of tensile and compressive strains obtained for unfavorable evolution cases (Figures 6, 8). Physically, this means that while the bulging wall tangentially expands, it is radially squeezed under the effect of the internal pressure. Under this conditions, the aneurysm is likely to become thinner, increasing the risk of aneurysm rupture. On the contrary, in absence of internal pressure on the cylinder, the strain vectors show no dominant direction (Figures 13a,c), also matching with our results for aneurysms with favorable evolution (Figures 5, 7).

Besides, there is also the need to evaluate the strains more objectively and globally to correlate them with the long-term evolution of the patient. For that purpose, we have computed the PCA for each patient tensile and compressive strain fields, obtaining 3 principal components per field that define the principal directions of the strains and that are used to perform a ROC-AUC analysis. Applying PCA to extract the global deformation pattern allows discarding noise from the deformation fields that may come from small errors in the segmentation or registration processes. When using tensile features alone to perform the ROC-AUC analysis, a mean AUC of 72.8% is obtained, which reflects that tensile strains are able to capture the differences between favorable and unfavorable evolution aneurysms. Regarding the compressive strain features, even through visual inspection the relation between compressive strain directions and patient evolution is less clear than the corresponding relation with tensile strain directions. This is confirmed by the mild performance of the classification based on the compressive features alone. Nevertheless, the ROC-AUC analysis using tensile and compressive features together yields an AUC of 88.6%, which confirms that these compressive strains contain important physical information about the wall mechanics and the evolution of the aneurysm, according to the aforementioned analogy with the cylinder stress theory.

Overall, a good correlation has been observed between the principal components of tensile and compressive strain fields together and long-term patient prognosis. This suggests that the strain information directly extracted from the CTA images is able to capture the biomechanical behavior of the aneurysm and it opens up the opportunity to further investigate a new way to evaluate aneurysm biomechanics directly from the image, instead of using finite element modeling and simulation. These models are sometimes difficult to calculate, which limits their applicability in the clinical routine, and require the assumption of some mechanical properties that are not precisely measurable *in-vivo*. With our proposed approach no assumption is required, and the proposed methodology could be easily translated into an intuitive semi-automatic workflow to assist the clinicians during the follow-up of patients.

Furthermore, the proposed principal components, treated as features, set the basis for possible future imaging biomarkers that may be used in clinical practice to assess patient prognosis and to enable informed decision making after an EVAR intervention, especially in difficult uncertain cases. These may include endotension cases or patients with visible endoleaks that do not shrink after the intervention. Besides, finding specific strain patterns depending on the endoleak type may also be valuable in order to better characterize the prognosis of the patient, which we consider an interesting future line of research.

The results are encouraging, but some limitations need to be addressed in order to make our approach translatable to the clinical practice. Firstly, errors in the segmentation of the different CTA structures, as well as in the registration pipeline, may subsequently produce errors in the computed final deformation fields. The influence of these errors in the analysis of the strain fields is partly overcome by using global principal component analysis, but a detailed evaluation of its potential impact should be assessed and minimized in a future stage. Similarly, the differences in the image spatial resolution may also influence the output strain fields, since during the registration process some information may be lost or interpolated when registering an image with a reference scan with a different image spatial resolution. The extent to which these variations alter the final strain fields should also be studied. On the other hand, our dataset is mostly composed of men, and thus the strain patterns may be different for women. Even if AAAs are about four times less common in women than in men, those who develop an AAA seem to have a worse prognosis than men (van de Luijtgaarden et al., 2017). Hence, a gender-based analysis should be carried out in order to find patterns associated to the sex of the patient. Furthermore, the evaluation of the proposed methodology with a larger image database, as well as improving the level of automation, is the aim of our future work.

## Data Availability Statement

The raw data supporting the conclusions of this manuscript will be made available by the authors, without undue reservation, to any qualified researcher.

## Ethics Statement

The studies involving human participants were reviewed and approved by Comité Ético de Investigación Clínica del Área Sanitaria de Gipuzkoa. According to the laws of our committee, since it is an anonymized image-based retrospective study, no informed consent is required.

## Author Contributions

KL-L developed the methodology, run some experiments, and wrote the manuscript. IG run some of the experiments. AG supervised the work from a clinical perspective. CC and JN provided their knowledge on the biomechanics domain. IM gave support with his knowledge on medical image analysis of abdominal aortic aneurysms. GP and MG provided methodological guidance and reviewed and revised the manuscript.

### Conflict of Interest

KL-L, IG, CC, and IM are employed by Vicomtech. The remaining authors declare that the research was conducted in the absence of any commercial or financial relationships that could be construed as a potential conflict of interest.
